# Barriers and enablers to implementing interprofessional primary care teams: a narrative review of the literature using the consolidated framework for implementation research

**DOI:** 10.1186/s12875-023-02240-0

**Published:** 2024-01-12

**Authors:** Amy Grant, Julia Kontak, Elizabeth Jeffers, Beverley Lawson, Adrian MacKenzie, Fred Burge, Leah Boulos, Kelly Lackie, Emily Gard Marshall, Amy Mireault, Susan Philpott, Tara Sampalli, Debbie Sheppard-LeMoine, Ruth Martin-Misener

**Affiliations:** 1 Maritime SPOR Support Unit, 5790 University Avenue, Halifax, Nova Scotia B3H 1V7 Canada; 2 Department of Family Medicine, Dalhousie University, Halifax, Nova Scotia Canada; 3 Building Research for Integrated Primary Care, Halifax, Nova Scotia Canada; 4 Nova Scotia Department of Health and Wellness, Halifax, Nova Scotia Canada; 5 Nova Scotia Health, Halifax, Nova Scotia Canada; 6 Faculty of Nursing, University of Windsor, Windsor, Ontario Canada; 7 School of Nursing, Dalhousie University, Halifax, Nova Scotia Canada

**Keywords:** Interprofessional teams, Primary care, Consolidated framework for implementation research, Implementation, Access to care

## Abstract

**Background:**

Interprofessional primary care teams have been introduced across Canada to improve access (e.g., a regular primary care provider, timely access to care when needed) to and quality of primary care. However, the quality and speed of team implementation has not kept pace with increasing access issues. The aim of this research was to use an implementation framework to categorize and describe barriers and enablers to team implementation in primary care.

**Methods:**

A narrative review that prioritized systematic reviews and evidence syntheses was conducted. A search using pre-defined terms was conducted using Ovid MEDLINE, and potentially relevant grey literature was identified through ad hoc Google searches and hand searching of health organization websites. The Consolidated Framework for Implementation Research (CFIR) was used to categorize barriers and enablers into five domains: (1) Features of Team Implementation; (2) Government, Health Authorities and Health Organizations; (3) Characteristics of the Team; (4) Characteristics of Team Members; and (5) Process of Implementation.

**Results:**

Data were extracted from 19 of 435 articles that met inclusion/exclusion criteria. Most barriers and enablers were categorized into two domains of the CFIR: Characteristics of the Team and Government, Health Authorities, and Health Organizations. Key themes identified within the Characteristics of the Team domain were team-leadership, including designating a manager responsible for day-to-day activities and facilitating collaboration; clear governance structures, and technology supports and tools that facilitate information sharing and communication. Key themes within the Government, Health Authorities, and Health Organizations domain were professional remuneration plans, regulatory policy, and interprofessional education. Other key themes identified in the Features of Team Implementation included the importance of good data and research on the status of teams, as well as sufficient and stable funding models. Positive perspectives, flexibility, and feeling supported were identified in the Characteristics of Team Members domain. Within the Process of Implementation domain, shared leadership and human resources planning were discussed.

**Conclusions:**

Barriers and enablers to implementing interprofessional primary care teams using the CFIR were identified, which enables stakeholders and teams to tailor implementation of teams at the local level to impact the accessibility and quality of primary care.

**Supplementary Information:**

The online version contains supplementary material available at 10.1186/s12875-023-02240-0.

## Background

Interprofessional primary care teams are a team-based approach to the delivery of primary care. The practitioners in these teams can vary, but typically include one or more family physicians and nurse practitioners (NPs) as well as one or more other healthcare providers, such as nurses, social workers, and pharmacists [1]. The use of team-based approaches can enhance access to primary care by reducing wait times, improving coordination of care, making more appropriate referrals, and reducing duplication of services and emergency department visits [2–4]. Interprofessional primary care teams can also reduce unnecessary use of resources, improve accessibility and patient satisfaction [5–7], improve chronic disease prevention and management, and reduce burnout among primary care team members [8–11].

Although this approach to the delivery of primary care has been implemented across Canada and world-wide as a means of increasing the accessibility and quality of primary care [12–15], the speed of implementation has not kept pace with current demand given increasing population size and complexity of patient care needs in many countries [16–18]. Access to primary care in Canada is now said to be in crisis [19–21], as a growing number of Canadians do not have a regular primary care provider, which has been further exacerbated by the COVID-19 pandemic [22]. Although there is an increased call for implementing interprofessional primary care teams in Canada [23] as a means to improving the primary care system, these teams are not the norm, and progress has stalled [20]. The way teams have been implemented has also varied, with increased panel sizes and clinician capacity not always being an outcome [24]. Given the potential of interprofessional primary care teams to improve primary care capacity, this research aimed to identify barriers and enablers to implementation to produce evidence that can be tailored to support new and existing teams. To do this, we used the Consolidated Framework for Implementation Research (CFIR) [25] to categorize and describe these barriers and enablers to team implementation.

## Methods

### Search and screening

Given the breadth of literature on this topic, our narrative review focused on qualitative, quantitative, or mixed-methods literature syntheses or systematic reviews, while also including high quality primary studies that met inclusion criteria (Table 1). The search strategy was developed and implemented in consultation with a health research librarian. The search was not designed to be systematic in nature, but rather was adaptive and iterative in order to best capture relevant studies. Search terms included keywords such as *primary health care, physicians, primary care, primary care nursing, general practitioners, general practice, family practice, physicians, medical home, collaboration, team/team-based, co-located, and barriers and enablers/facilitators.* Literature from other countries that were similar in context were included. The search was executed in the Ovid MEDLINE database to identify peer-reviewed articles.

**Table 1:** Inclusion/exclusion criteria

**Inclusion Criteria**				
Primary care (PC)	AND	Team	AND	Co-located
General practice		Collaboration		In same office/clinic
Family practice		Nurse		One physical location
Family doctor		Psychologist		The collaborating partner does not have to be ONLY or always in the PC office/clinic but must provide services there (e.g., a surgeon spends one day per week at a PC office/clinic treating patients; the remainder of time they are in the hospital). This meets our definition.
General practitioner		Social worker		
GP		Partner		
General practice		Shared care		
Medical home		Allied health professional		
**Exclusion Criteria**				
Solo/individual GP/family doctor/etc.	OR	No team or collaborative/ partnering aspect. Partnering/ team must be with other care providers	OR	Care or services are provided at different locations.
Physicians only				
Nurses only				
Any lone/individual provider (e.g., a psychologist only, a nurse practitioner only, a social worker only etc.)				
Inpatient/outpatient care				
Homes for aged				
Hospital clinic/care				
Community care/clinic				

The search was initially executed in 2019 and updated in July 2021 and December 2022. Grey literature was identified through Google searches and hand searching of health organization websites, focusing on Canadian health organizations and well-known American health organizations (Appendix I). Only articles available in English were included as the team members could only speak/read in English. Articles were screened using the inclusion/exclusion criteria outlined in Table 1. Screening was conducted by a group of three research team members (LR, SM, AG) using Covidence™ online, a literature review management software that helps to streamline the review process (Veritas Health Innovation, Melbourne, Australia available at www.covidence.org). Following the search, all identified records were uploaded into Covidence and duplicates removed. All screening processes from title/abstract were completed in Covidence. All included articles were independently screened (title and abstract followed by full text review) by two or three individuals noted above (LR, SM, AG). Conflicts were discussed amongst the three reviewers to establish agreement.

For the purposes of this review, teams were considered to meet the definition of an interprofessional primary care team if they included at least two different healthcare provider types that were co-located in a primary care practice setting [26, 27]. Although other definitions may exist, this definition was chosen as it meets the definition identified locally and is used to support implementation of teams [28].

### Extraction

The five domains from the CFIR were renamed to reflect this study more directly (Table 2). The CFIR Intervention domain was renamed *Features of Team Implementation and Effectiveness*, the Outer Setting domain was named *Government, Health Authorities and Health Organizations*, Inner Setting was named *Characteristics of the Team*, Characteristics of Individuals was represented by *Characteristics of Team Members*, and Process by *Features of the Process of Implementation*.

**Table 2:** CFIR domains, domain relabeled for teams and description, adapted from Damschroder et al. 2009 [25]

CFIR Domains	Relabeled	CFIR constructs	CFIR sub-constructs	Description
**I: Intervention Characteristics**	Features of Team Implementation and Effectiveness	8	0	This domain contains eight constructs related to beliefs, perceptions, and characteristics of the intervention, which is defined as implementation or creation of the team.
**II: Outer Setting**	Government, Health Authorities and Health Organizations	4	0	This domain is defined as the collaborative family practice team and includes four constructs.
**III: Inner Setting**	Characteristics of the Team	5	9	This domain refers to the practice (i.e., the entity of a practice, which includes the health professionals, administration, managers, etc.), and consists of five constructs.
**IV: Characteristics of Individuals**	Characteristics of Team Members	5	0	This domain refers to any individuals working within a team and includes five constructs
**V: Process**	Features of the Process of Implementation	4	6	This domain refers to the implementation of the team and includes four constructs.

Extraction of data was completed by two research team members for each article (LR, SM, AG, AM). Items were coded into the most appropriate CFIR *domain*, *construct, or sub-construct* using the original version of the CFIR [25]. An updated version has recently been released, and constructs can be mapped onto the updated version if needed [29]. Conflicts related to the categorization of items into the CFIR constructs were flagged and discussed by the group with the principal investigator (RMM) to establish agreement.

### Content analysis

We used deductive content analysis using the CFIR to categorize extracted barriers and enablers [25, 30]. Themes were then identified through inductive content analysis within these domains [31]. Lastly, to further enable identification of patterns within the included articles, we used summative content analysis to calculate the number of articles that addressed a specific CFIR domain, and the number of barriers and enablers found [30]. This was done because the frequency of barriers and enablers does not always represent unique items. For example, if a similar barrier was identified in multiple articles it was extracted in each instance and included separately in the frequency calculation so as not to introduce bias around identifying whether each barrier or enabler was unique. The number of articles which discussed barriers and enablers was calculated, as well as the number of barriers and enablers.

### Degree of overlap

Given the potential risk of bias due to inclusion of primary studies in more than one review [32], we calculated this review’s degree of overlap with previous reviews using the Corrected Covered Area (CCA) measure [33]. This provides an indication of the extent of overlap for primary studies in a review of reviews and can also be used to further understand differences in methodology and outcomes across the literature [34]. A CCA value of ≤ 5% indicates a slight overlap, 6–10% moderate overlap, 11–15% high overlap, and values ≥ 15% indicate a very high overlap [33].

## Results

**Fig. 1:**
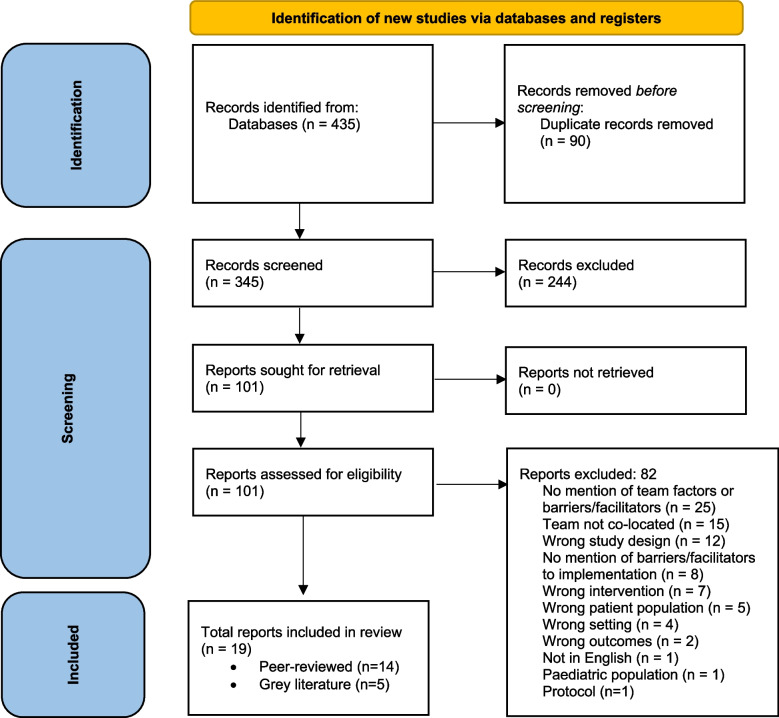
Flow Diagram of screening process

The searches (2019, 2021, 2022) yielded 435 records of which 90 duplicates were removed, and a further 244 excluded through title and abstract screening (Fig. 1). After full-text screening of 101 articles, 82 articles were excluded as they did not meet the inclusion criteria, leaving 19 articles for data extraction. For a detailed depiction of the screening and selection process, refer to the PRISMA diagram in Fig. 1. Of the 19 articles included in the review (Table 3), 16 used review methodology, which encompassed 441 primary studies, with a median of 26.5 articles included in each review (range 9-100). Most of these primary studies were included in one review (*n* = 411) and the remainder in two (*n* = 26), or three (*n* = 5) reviews. The degree of overlap calculated by the CCA measure was 0.53%, indicating that this review has a very slight degree of overlap with previous reviews.

**Table 3:** Description of literature included in review

Author	Yearss	Geographic location	Design
Dinh/Conference Board of Canada [35]	2012	Canada, Australia, England, Netherlands	Literature review
Virani et al. [36]	2012	Canada: Nationally distributed	Scoping review
Registered Nurses' Association of Ontario [37]	2013	Canada: Ontario	Systematic review
Dinh/Conference Board of Canada [38]	2014	Canada & United States of America (USA)	Survey, Interviews, & Literature review
Morgan et al. [39]	2015	Canada: Ontario, Quebec, Saskatchewan; Australia, Sweden, United Kingdom (UK).	Integrative review
Wranik et al. [40]	2015	Canada: Alberta, Manitoba, Nova Scotia	Scoping review of published & grey literature; Stakeholder interviews/survey.
Mulvale et al. [41]	2016	Canada, Spain, UK, USA, Puerto Rico	Systematic review
O'Reilly et al. [42]	2017	Canada, USA, UK, Australia, New Zealand, Sweden, France, Spain, Netherlands, Brazil, South Africa	Integrative review
Bentley et al. [43]	2018	Australia	Online survey & interviews
Grol et al. [44]	2018	Netherlands	Focus groups, Interviews
Russell et al. [45]	2018	USA, Canada, Australia	Collaborative reflexive deliberative approach
Sorensen et al. [46]	2018	Norway	Scoping review
Wranik & Haydt [47]	2018	Canada: Manitoba, Nova Scotia, Alberta	Interviews, Policy documents
Levis-Peralta et al. [48]	2020	USA, Canada, Europe (UK and Netherlands), Asia (China), and the Middle East (Oman)	Scoping review
McNaughton et al. [49]	2021	Australia, Brazil, Canada, New Zealand, The Netherlands, South Korea, Sweden, USA, and United Kingdom	Scoping review
Dankoly et al. [50]	2021	Australia, USA, Ireland, UK, and Canada	Systematic Review
Rawlinson et al.[51]	2021	International (USA, UK, Canada, Australia, Sweden, Switzerland, Germany, Ireland, Spain, Puerto Rico, France, Netherlands, Brazil, Republic of South Africa, Lithuania, Norway, Denmark, Belgium, Iran, Malaysia, Scotland, Wales, Cuba, Nepal, Bangladesh, Indonesia, Tanzania, Nigeria, Thailand, Peru, Columbia, Finland)	Overview of reviews
Holmes and Change [52]	2022	USA and Canada	Integrative review
Perron et al. [53]	2022	Canada, Australia, New Zealand, USA, Norway,	Scoping review

The number of articles that addressed a specific CFIR domain, and the number of barriers and enablers found within each of the sub-constructs or research themes identified within the CFIR domains was calculated (Table 4). Fewer barriers and enablers (frequency [f] = 18) were identified that related to implementation of interprofessional primary care teams compared to most of the other CFIR domains (f = 42, 200, 18, and 34 in *(I) Features of Team Implementation and Effectiveness*; *(II) Government, Health Authorities and Health Organizations*; *(III) Characteristics of the Team*; *(IV) Characteristics of Team Members*; *(V) Features of the Process of Implementation*, respectively). More detail on the barriers and enablers not fully described below can be found in Table 5.

**Table 4:** Number of articles reporting barriers and enablers within CFIR domains, constructs, and sub-constructs

CFIR Constructs	CFIR Sub-constructs or research identified themes (denoted by*)	# of articles	# of barriers	# of enablers
**CFIR Domain I: Intervention Characteristics**				
A Intervention Source		0	0	0
B Evidence Strength & Quality		2	0	2
C Relative Advantage		0	0	0
D Adaptability		0	0	0
E Trialability		0	0	0
F Complexity		0	0	0
G Design Quality & Packaging		2	1	2
H Cost		8	6	7
**CFIR Domain II: Outer Setting**				
A Patient Needs & Resources		4	2	7
B Cosmopolitanism		8	1	11
C Peer Pressure		0	0	0
D External Policy & Incentives	D.1. Funding Models & Compensation*	10	6	5
	D.2. Government & Regulatory Policy*	7	3	3
	D.3. Education*	3	1	3
**CFIR Domain III: Inner Setting**				
A Structural Characteristics	A.1. Team size & composition*	8	3	11
	A.2. Governance*	7	4	3
	A.3. Team Organization & Coordination Supports*	7	3	6
B Networks & Communications	B.1. Communication Tools & Technology*	11	1	19
	B.2. Formal Communication*	10	0	15
	B.3. Informal Communication*	9	2	10
	B.4. Role Clarity & Relationships*	14	14	14
C Culture	C.1. Trust & Respect*	7	2	2
	C.2. Shared Purpose & Identity*	6	4	5
	C.3. Power & Hierarchy*	10	9	8
D Implementation Climate	D.1. Tension for Change	1	0	1
	D.2. Compatibility	0	0	0
	D.3. Relative Priority	0	0	0
	D.4. Organizational Incentives & Rewards	2	0	2
	D.5. Goals & Feedback	5	1	8
	D.6. Learning Climate	7	4	10
E Readiness for Implementation	E.1. Leadership Engagement	6	4	5
	E.2. Available Resources	12	10	18
	E.3. Access to Knowledge & Information	2	0	2
**CFIR Domain IV: Characteristics of Individuals**				
A Knowledge & Beliefs about the Intervention		6	3	5
B Self-efficacy		0	0	0
C Individual Stage of Change		0	0	0
D Individual Identification with Organization		0	0	0
E Other Personal Attributes		6	6	4
**CFIR Domain V: Process**				
A Planning		2	1	2
B Engaging	B.1. Opinion Leaders	4	3	1
	B.2. Formally Appointed Internal Implementation Leaders	3	0	3
	B.3. Champions	7	0	7
	B.4. External Change Agents	0	0	0
	B.5. Key Stakeholders	0	0	0
	B.6. Innovation Participants	2	3	2
C Executing		0	0	0
D Reflecting & Evaluating		7	1	11

**Table 5:** Consolidated list of barriers and enablers to implementation of teams using the CFIR

CFIR Domains	Enablers	Barriers
**I: Intervention Characteristics**	Buy-in from medical professional organizations Good data and research to understand impact of changes in system Neutral funding models that link funding to activities of whole team on a per patient basis Independent income generation, not dependent on their activities or those of colleagues Resourcing and funding for interprofessional practice and related initiatives	Unstable, inadequate, or lack of long-term funding or reimbursement models Space & equipment covered by income of a specific provider
**II: Outer Setting**	Client-centered approaches (i.e., assessing patient/community characteristics and needs) Involving patient and family in care planning and delivery Patients willing to receive care from teams. Multi-component models that involve patient education, systematic follow-up, medication adherence GP networking in community to establish contacts with community partners (e.g., social services, hospitals) Managers supporting integrating care (e.g., care coordination, connecting to social services, nursing homes, prevention resources) Inter-organizational collaboration, including service integration and coordinating care for patients with complex needs GPs in alternate payment plans (APPs) may be more incentivized to participate in collaborative activities than fee for service plans. Health professional regulatory bodies incorporating interprofessional competencies into licensing requirements Incorporating interprofessional education into academic curricula for healthcare professional programs, pre- and post- licensure Graduate level education for advanced practice nurses System-level collaboration and policies (i.e., legislative and regulatory reforms) which may set targets for interprofessional care or introduce non-physician professionals into teams	Lower compensation and benefits for teams compared to hospitals and private sector results in poor recruitment and retention Different remuneration systems for different professionals (e.g., referrals from GPs vs. NPs) When funding or compensation does not facilitate participation in team (e.g., meetings discussing patients) Salaries that originate from different funding sources Fee for service payment models, which reward interprofessional isolation. Top-down policies that require physician authority or decision-making Team members lack competency in interprofessional collaboration due to lack of/inadequate interprofessional training Difficulty in engaging with wider community in rural/remote areas when practitioners are new
**III: Inner Setting**	Move away from physician-driven care; include nurse practitioners on team Adopting a “whole system” approach by involving non-clinical staff and clerical staff on team Ensure there is an established team leader/manager responsible for managing and facilitating collaboration and day-to-day activities Single-handed governance structures, in place of a partnerships, are positively associated with team climate Clinics operating under a board of directors Integrating both bottom-up and top-down governance associated with heightened efficiency and coordination Developing new organizational infrastructure crucial for care delivery Tech supports (e.g., EMRs, computerized message & booking, telehealth) facilitate collaborative decision making and information sharing Standardize documentation and tools (e.g., integrated care pathways, common patient charts, interprofessional care plans) Encourage information sharing, task delegation, and supportive communication through: weekly scheduled interprofessional team meetings, frequent and reciprocated ad-hoc communications (e.g., clinic huddles) Meetings include procedures for negotiation, decision making and conflict management and resolution Clearly defining roles and understanding roles and respective scopes of practice Set interprofessional guidelines (e.g., referral mechanisms between members) Interprofessional case conferences allows opportunity to collaborate Non-hierarchical organizational structure that encourages equality, mutual respect, low levels of conflict, willingness to cooperate and collaborate Balance between group culture, hierarchy and focus on efficiency and achievement Balanced power relationships through shared leadership, decision making, authority and responsibility Identify and adjust power imbalances to build mutually supportive workplaces Financial incentives based upon unique collaborative care demands (e.g., after-hours services, compilation of care plans) Feeling supported and formally recognized for performance Opportunities for all staff to receive bonuses based upon target achievement A clear vision and well-defined goals that have been collectively identified contribute to a shared sense of purpose Processes for group decision making and problem solving promote shared purpose amongst the team Colocation leads to greater mutual understanding, increased role clarity and superior care delivery Educating staff in interprofessional care on the job (e.g., social and organizational training to mitigate power dynamics and training on co-workers’ roles) Offering learning opportunities and leadership training courses to support collaboration Clearly explained team processes, policies, and procedures as well as accessible and intuitive documentation	Lack of clear/inadequate leadership, and system-level leadership Ambiguous roles, lack of understanding of the knowledge and skills of different professionals, and concerns about professional scope and liability Lack of training or experience required to evolve into facilitators of collaboration Physical separation creates a symbolic barrier and reinforces perceived divisions Insufficient workspace or profession-specific spaces negatively impact communication, workflow, and team cohesion Lack of training or educational opportunities Insufficient time in the day to engage in and share reflections and learnings, instill a trusting environment Insufficient human resources impact the implementation of initiatives to improve collaborative care
**IV: Characteristics of Individuals**	Belief in, or positive attitude towards, the concept of collaboration The ability to be flexible in one’s professional role within the team GPs accommodate the new skill mixes on a team and acknowledge the potential benefit of non-physician/patient interactions Collaborative skills possessed by individuals within the team	Opposition or disagreement among team members on the potential value of interprofessional initiatives and education, and the impact on patients Opposing interests, values, and beliefs and interprofessional conflict Concern or territoriality around one’s role within the team, with a shift in attitude needed to allow all appropriate team members to have meaningful patient interactions
**V: Process**	Plan human health resources in a manner that encourages collaboration and coordination Establish human resource plans that allow time for staff to participate in interprofessional activities Reduced team turnover to optimize growth To foster future collaboration, allow opportunities for students from different professions/programs to engage with one another Promote greater interprofessional networking Management structures and system level foundations that are explicitly collaborative and support local leadership and team development & processes Engage and develop interprofessional leaders among the point-of-care health professional Developing and having team champion(s) and facilitators within the team to integrate team actions, facilitate team building External accountability like focusing on quality through audits or other processes and motivate a collaborative approach to problem solving Monitoring and evaluation are a method to overcome system level barriers to interprofessional communication Team members reflecting on their practice and sharing informal feedback with colleagues about their interprofessional work	Limited human resource planning Physician reluctance to collaboration Reluctance of patients to see multiple providers Difficulty reporting relevant outcomes measures of interprofessional education and practice

### Domain I - intervention characteristics: features of team implementation (f = 18)

Most of the data in this domain was related to the *Cost construct* (f = 13), and focused on which funding arrangements were more likely to encourage collaboration than others [37]. Aspects of cost discussed included issues with inadequate funding and insufficient reimbursement [50, 51], Resources, including the availability of human resources, the stability of staffing, and user friendly information systems, were also discussed [48, 49].

Enablers related to the *Evidence Strength & Quality construct* were also identified (f = 2), including the need for high quality data and research to understand the current status and impact of interprofessional primary care teams in the Canadian system [36], such as the need for new members like NPs, which requires buy-in from healthcare professional organizations [45].

### Domain II - outer setting: government, health authorities, and health organizations (f = 42)

Several constructs within this domain were identified as important to the functioning of interprofessional primary care teams. Most of these barriers and enablers were grouped into the *External Policy and Incentives* construct (f = 21), specifically within the sub-construct of funding models (f = 11), which are typically set by governments or other organizations distinct from the team itself [51]. A common enabler was that interprofessional practitioners indicated preference for salary versus fee-for-service models [36, 37]. As noted above, studies found that physician remuneration with fee-for-service models impedes team implementation [40, 47] by siloing care [37], rewarding professional isolation [45], and discouraging participation in interprofessional education [38]. However, the capacity to maximize billing to offset costs related to team-based care and the models that exist for staff compensation (e.g., patient per month, salaried, hourly) was also identified as an enabler [48]. Payment models affect collaboration, for example, those in alternative payment models may be incentivized to participate in team meetings [35] compared to fee-for-service [44]. Physicians paid through fee-for-service models curbs financial incentives to participate in shared decision-making with other team members, given that there is no billing codes associated with this activity [38]. Financial hierarchy among providers working together in interprofessional primary care teams is another barrier such that a physician’s activities may determine the funding available to pay other healthcare providers [40]. Further, when compensation and benefits for primary care team positions are not competitive with those in hospitals and/or the private sector, recruitment and retention of qualified personnel is hindered [35].

Government-led barriers and enablers (f = 6) of interprofessional primary care teams were also identified within this construct, with an enabler being the allocation of funding for the implementation of interprofessional primary care teams and developing policy that supports interprofessional collaboration [37]. An example was system-level reforms to expand teams by adding health professionals such as pharmacists, dieticians, and social workers [45, 49]. In contrast, legislation that requires physicians to sign off on the actions of other providers perpetuates interprofessional power differences [45]. The remainder of the barriers and enablers within this construct were related to education (f = 4). More specifically, interprofessional education [37], knowledge of interprofessional competencies [36, 38], and incorporation of these competencies into licensing requirements [37] enable interprofessional collaboration that in turn supports team functioning.

Enablers related to the *Patient Needs and Resources construct* (f = 7) included having a multi-component model of care including: patient, family, and caregiver education; systematic follow-up; and medication adherence support (beyond, for example, diagnosis and treatment) [37]. Person-centered care, along with culturally safe and acceptable practices [49], as well as the importance of understanding patient populations with socioeconomic needs [52] were identified as enablers. A related enabler identified was enhanced team awareness of patient population characteristics and needs, possibly through the use of community needs assessments [36]. Patient willingness to receive care from teams, as well as involvement of patients as decision-makers in care planning and delivery were also identified as important [36].

With respect to collaboration across levels of the system, within the *Cosmopolitan* construct (f = 12), working relationships between healthcare professionals located in different practice settings to coordinate care for patients, particularly when patients have complex needs were deemed as important [46]. Supporting integration and coordination of care among team members in the practice, typically through the team manager role [44, 49], while being able to exchange clinical and billing information across providers or practices also improves coordination of services with the community [48]. Practice-based linkage to the community and community services [48], especially for rural and remote areas [53] was discussed. Maintenance of a broad awareness of services available external to the practice – such as hospitals, nursing homes, social, and community services, along with knowledge of the necessary processes for accessing such services were also enablers [44, 49].

### Domain III - inner setting: characteristics of the team (f = 200)

Many articles identified barriers and enablers related to *Structural Characteristics* (f = 30), with the bulk related to team size and composition (f = 14), which was identified as both a barrier of and enabler to interprofessional collaboration and teamwork [41]. Interprofessional primary care teams that are too large can impede functioning [40, 41, 47] and effectiveness [35]. However, smaller teams may not be able to provide the accessibility, continuity, and quality of care patients need [35]. The presence of NPs on teams was identified as a feature that supports successful implementation of interprofessional primary care teams to meet the needs of a patient population [36]. Other roles described as important to team composition included having specialist informatics staff (e.g., data manager), client navigators, and case managers [48, 49]. Enablers related to team organizational supports (f = 6) (e.g., clear business plan, a governance mechanism, work place policies) [36] and taking a ‘whole-system’ approach, included non-clinical staff such as human resources and social services, which enable interprofessional primary care team implementation [35]. The make-up of the team, including formalized partnerships and/or co-located spaces between providers were identified as enablers [51]. Difficulty with staff turnover [52], as well as recruitment and retention of health professionals, including in rural and remote areas, were identified as barriers [53].

With respect to governance (f = 7), models that include a board of directors governing the team have demonstrated consistently high team climate scores [45]. When such governance is in place, this may aide the team in sustaining transformative changes through the established leadership, policies and procedures that support the team [35]. Conversely, top-down leadership approaches [49] - specifically in privately-owned practices governed by physicians who make critical organizational decisions and who receive all practice profits as other staff are typically paid by salary [45] - can be barriers to implementation.

The *Networks & Communications construct* was one of the most commonly identified across the literature (f = 75) with electronic medical and/or health records (EMRs, EHRs), computerized messaging, and telehealth [35, 42, 48] discussed as enablers to team communication. Barriers (f = 17) include technologies not designed for recording interprofessional work [43] and disagreement among team members around use of care plans [36]. Formal communication mechanisms (f = 15), such as regularly scheduled team meetings, case conferences, and huddles also enable interprofessional primary care team implementation [35, 37, 38, 41, 46, 48, 49]. Such meetings are identified in included articles as opportunities to collaborate about patient care, discuss team schedules and plans [35], and gain an understanding of team members’ roles and priorities [37]. In general, formalizing communication procedures supports collaboration between all healthcare providers [46]. Informal communication enablers (f = 10) include unplanned communication approaches (e.g., hallway conversations) [39] and may facilitate shared decision making and collaboration [42], but are insufficient on their own [35]. The importance of role clarity (f = 14) among team members was discussed regularly [42, 48–50] with many studies pointing out the frequency and negative impact of the lack of role clarity [35, 42], including inadequate knowledge of other team members skillsets and scopes of practice [35, 53]. Barriers that prevent role clarity (f = 14) can be attributable to gaps in knowledge and/or misunderstanding of roles among team members [35] and inadequate communication about provider roles in educational programs [40].

*Culture* within the team was also a frequent subject of study (f = 30). Within trust and respect (f = 2) feeling acknowledged, and being open to others’ perspectives [42, 46], are linked with role clarity, and regarded as essential for interprofessional primary care team implementation and collaboration [35, 42, 46, 49]. Shared purpose and identity (f = 5) are important aspects of culture that promote team implementation [40, 43] and facilitate collaborative organizational change through a positive, motivating culture [49]. In contrast, a focus on throughput or productivity [49], professional silos [40], and issues interfering with team cohesion [36] create barriers (f = 4) to team implementation. Balanced power relationships (f = 8) among team members occurring through shared leadership, decision making, authority, and responsibility enable team implementation [37]. Hierarchical relationships in the team [38, 49] and physician hierarchy in particular, are barriers (f = 9) to team implementation [42, 46, 47]. Interprofessional primary care teams have a sense of equality among members and understand and rely on their individual strengths and capabilities [36].

Within *Implementation Climate* (f = 26), having external stakeholders supports collaboration with external partners and thus supports the implementation of the intervention (i.e., the interprofessional primary care team) itself [49]. Within the team itself, having a clear, properly communicated, and coordinated team vision or shared goals and objectives enables team implementation [36, 40, 41, 49]. When shared goals are explicitly communicated it adds to the sense of common purpose and improves the buy-in of team members with the collaborative process [40]. Support and innovation within the team [41], in addition to having dedicated time and support for collaborative learning and practice of interprofessional practice skills [48, 49] facilitates collaboration. Team implementation is also enabled by payment incentives for after-hours services and for care plan compilation, capitation models, and salary support for leadership and administrative roles [45].

Lastly, within this domain and the *Readiness for Implementation construct* (f = 39), is the importance of leadership [36, 38, 52], including at the system-level, that promotes and supports collaboration [46]. Leadership courses [37], interprofessional education on the job [37], and teamwork training [36, 40, 42] may reduce team turnover to optimize growth of teams [37]. Additionally, having time and resources (e.g., technological supports) and equal professional development opportunities amongst team members are important enablers [48, 49]. A key measure of the readiness for implementation is the degree to which available physical space allows for satisfactory co-location of the team [42, 48]. This was identified as a factor that can result in greater mutual understanding, increased understanding of one another’s roles, and enhanced delivery of care [37, 44]. Insufficient overall, and designated space for each provider negatively impacts communication, workflow, and team integration as this may inhibit individuals from physically working together in a shared space at the same time [37, 47, 48].

### Domain IV - characteristics of individuals: characteristics of team members (f = 18)

Compared to other CFIR domains, fewer papers discussed specific individual characteristics that were important to implementation (f = 18). Some barriers and enablers were grouped into *Knowledge and Beliefs about the Intervention* (f = 8), with positive views toward collaboration and collaborative care models [41, 51–53] as an enabler whereas conflicting interests, values, beliefs or other interpersonal conflicts were identified as barriers [37]. *Other Personal Attributes* (f = 10) identified flexibility – particularly in one’s role – as an enabler of team implementation [53], while concern about maintaining ownership over roles creates barriers to team implementation [36, 41].

### Domain V - process: features of the process of implementation (f = 34)

In *Planning* for the intervention (f = 3), health human resource planning (i.e., ensuring the right number and types of providers are in place to support patient care) to support collaboration and coordination of services were enablers of team implementation [36, 37]. Within the *Engaging construct* (f = 19), the enabling roles of those who can take leadership responsibilities, integrate actions of the team, and provide a clear vision are important [44]. Developing and/or identifying interprofessional care champions [37, 41, 49] from within the team composition [42], is an enabler to team implementation. Systems that support organizational management and leadership [46], management structures that are collaborative, and offer regular feedback on team performance were identified as enablers of collaborative team implementation [39]. In recent literature, there were also barriers and enablers identified around the sub-construct *Innovation Participants* (f = 5), which spoke to the value of patient and community partnership and participation in the intervention [49]. Conversely, the reluctance of patients to see multiple providers was identified as a barrier [50]. Co-design of the practice environment and processes was an important enabler, while not including clients in the decision-making was identified as a barrier [49].

Lastly, within the *Reflecting and Evaluating construct* (f = 12) formal evaluation of team and collaborative care functioning was identified as an enabler [42], along with the ability to monitor performance and report measurable outcomes [49]. Informal feedback among healthcare providers about their interprofessional work and self-assessment and reflection on their own practice were also noted to be enablers of team implementation [37, 42].

## Discussion

The objective of this study was to identify, categorize, and describe barriers and enablers to primary care team implementation identified in the literature to support the work of existing and newly formed teams. The review identified 19 articles documenting barriers and enablers to implementation of interprofessional primary care teams. Much of the literature to date has focused on describing barriers and enablers in a specific context [48] or has been carried out by healthcare organizations with a specific viewpoint [36, 37]. The current review had an implementation focus and used the CFIR to categorize barriers and enablers. This work can be used as a basis to guide evidence-informed implementation of interprofessional primary care teams as they increasingly become a central focus of primary care reform in Canada [23].

Although reforms may differ across countries, they can be guided by 13 possible levers identified by the WHO as supporting primary care reform [54]. The choice of implementation actions should be guided by contextually relevant evidence in each country, with guidance suggesting action on some or all levers. These include actions such as political leadership and commitment – recognizing the importance of universal health coverage in providing equitable access to care, engagement of the community and stakeholders to identify problems and solutions - focusing on the primary health care workforce including quantity, competency and multiple disciplines, and models of care that promote primary care and integrated health. Many of these levers also align with the model of interprofessional primary care teams. Evidence supports interprofessional education as an approach to enable healthcare trainees to be open to collaboration and to become part of interprofessional teams [55]. This also facilitates understanding of other professionals’ roles/scopes of practice on teams, which was identified as a barrier in the current review.

Most of the information found in our review related to the barriers and enablers within the Characteristics of the Team (i.e., CFIR’s inner setting) and the Government, Health Authorities, and Health Organizations (i.e., CFIR’s outer setting) domains. Key characteristics of the interprofessional team that influenced team implementation included governance structures, formal and informal communication [35, 37, 38, 41, 46, 48, 49], power [37], and training [36, 40, 42]. Details relating to what constitutes an optimum size and composition of a team were unclear based on the literature reviewed. This finding is not unexpected as team size and composition would depend heavily on the context and circumstances of any given team, the needs of the population they serve, and population size. The main factors impacting the practice, organization, health authority, and government levels (i.e., CFIR’s outer setting) included professional remuneration [45], regulatory policy [36, 37], and interprofessional education [38].

Findings reiterate the stance that for interprofessional collaborative care to be carried out successfully, specific mechanisms need to be used to advance interprofessional practice, and this work cannot be done in silos. Commonalities across all domains included collaboration at varying levels of influence, whole-system approaches to governance structures and decision-making, dedication to interprofessional education and resources, and notably the impact of funding models.

Payment models that promote teamwork (e.g., salaried, alternative payment models) can aid in collaboration by focusing on team activities versus specific provider outputs as in more traditional fee-for-service funding models [40, 56]. Fee-for-service models were commonly cited as a barrier to team functioning, such that possible loss of potential income (e.g., when participating in team-based activity), negatively impacts the opportunity for shared-decision making and may also limit opportunities for interprofessional education [38]. However, distinct pathways of how to move away from a fee-for-service model or what an ideal model of compensation would look like to appeal to all providers were insufficiently described in the literature reviewed.

The scarcity of information about the practicalities of introducing team-based primary care as an intervention in healthcare systems is noteworthy and worthy of consideration for future research. This review is intended to aid in the development of strategies for effectiveness and growth of interprofessional primary care teams. Key messages to stakeholders in government and health authorities, team-level clinicians and managers, and healthcare educators and regulators are presented below and in the visual summary of findings (Appendix II).

### Key messages

Based on the enablers identified within the review to support team implementation in primary care, the following key messages for three stakeholder groups were identified.

#### Government and health authorities

- Design and implement funding models that link compensation to indicators of collaboration and team functioning in a manner that includes all team members.
- Ensure physical space allows for co-location of interprofessional primary care teams to promote mutual understanding, enable collaboration, and enhance care delivery.

#### Team-level clinicians and managers

- Commit to shared transformative leadership approaches, collaborative processes, and effective managerial support for change and conflict management.
- Implement technological tools to enable communication and facilitate information sharing (e.g., instant messaging EMRs) which are key to collaborative decision-making.

#### Health professional educators and regulators

- Implement policies, programs and resources that enable all team members to optimize their scope of practice and promote the development of non-hierarchical collaborative professional relationships.
- Establish pre- and post-licensure interprofessional education that addresses power and hierarchy to advance interprofessional collaboration and team implementation to improve healthcare delivery and experience.

### Strengths and limitations

This review included 19 articles focusing on the implementation of interprofessional primary care teams across various contexts and countries, 15 of which were existing reviews. Using the CFIR, we categorized this evidence according to key constructs, providing a logical structure to guide the development of implementation strategies. Use of theory to guide implementation enables factors of influence to be linked with appropriate intervention strategies and improves the generalization of findings through a common terminology [57]. This review will thus be useful for academia and primary care practice and policy. However, despite this strength, it is important to recognize that the existing literature has been influenced by the direction of governments and health service delivery organizations that have been implementing models of care as well as health professional organizations (e.g., nursing associations in this review) who have focused on the role of nurses in team-based care. As a result, the findings may be biased by this literature and its historical development.

Our review focused on co-located interprofessional primary care teams. In having this focus, it is possible we may have missed other types of collaborative care offered by alternative team configurations (e.g., teams located in community settings such as schools) especially important given the increased use of virtual care options through the COVID-19 pandemic. Additionally, for pragmatic reasons the search for this narrative review was limited to a single database (Ovid MEDLINE). This database was selected due to its comprehensive nature.

The CFIR was modified to suit the project based on team discussions, as it was designed to be tailored to the intervention design and context being studied [25]. Given the flexibility of the CFIR and overlap in how some barriers and enablers could be interpreted (i.e., into multiple domains), this may lead to differences in how the extracted data are interpreted, despite our efforts to reduce bias noted above. Also, the number of articles reporting each construct was reported, and the number of barriers and enablers extracted was summed (Table 4). However, these numbers do not capture a precise citation frequency. Rather, the value add is that this provides an indication of patterns and gaps in the literature.

## Conclusions

This review included a synthesis and qualitative organization of published literature, guided by the CFIR, to understand the barriers and enablers to the implementation of interprofessional primary care teams. Key influences identified included the importance of team-level leadership, adequate administrative and managerial resources, and a structured focus on communication, information sharing, and collaboration in a shared space. At the government and health authority level, there was a heavy focus on professional renumeration structures and policies (or lack thereof) to encourage collaborative team-based care. The results of the review may be useful to policymakers and health administrators seeking to change policy, and to primary care practices who are currently working in a collaborative team or looking to form one.

### Supplementary Information

**Additional file 1: Appendix-I.** Grey literature list of sources. **Appendix-II.** Infographic

## Data Availability

Data sharing is not applicable to this article as no datasets were generated or analyzed during the current study.

## Abbreviations

CCA: Corrected cover area
CFIR: Consolidated Framework for Implementation Research
EHR: Electronic health record
EMR: Electronic medical record
NP: Nurse practitioner
