# Supplementary material for: Barriers and enablers to implementing interprofessional primary care teams: a narrative review of the literature using the consolidated framework for implementation research
Source: BMC Prim Care. 2024 Jan 12;25:25. doi: 10.1186/s12875-023-02240-0 (PMC10785376; doi:10.1186/s12875-023-02240-0)

**Appendices**

**Appendix-I – Grey literature list of sources**

| **Organization** | **Website** |
| --- | --- |
| Government of Quebec | <https://www.quebec.ca/en/health/> |
| Government of New Brunswick | <https://www2.gnb.ca/> |
| Government of Saskatchewan | <http://www.sma.sk.ca/> |
| Government of Alberta | <http://www.health.alberta.ca/>  <https://open.alberta.ca> |
| Ministry of health and long-term care Ontario | <https://www.health.gov.on.ca/en/> |
| General Practice Services Committee (GPSC) British Columbia | <http://www.gpscbc.ca/> |
| Divisions of family practice (Northern British Columbia) | <https://divisionsbc.ca/northern-interior-rural> |
| British Columbia Medical Journal | [https://bcmj.org/gpsc/](https://bcmj.org/gpsc/new-gpsc-incentive-supports-family-doctors-implement-panel-management) |
| Health Sciences Association | <https://www.hsabc.org/> |
| Patient-Centered Primary Care Collaborative Organization | [www.pcpcc.org](http://www.pcpcc.org) |
| The Change Foundation | <https://www.changefoundation.ca/> |
| American Academy of Family Physicians (AAFP) | [www.aafp.org](http://www.aafp.org) |
| General practice services committee | <http://www.gpscbc.ca/> |
| Institute for Healthcare Improvement | <http://www.ihi.org/> |
| Canadian Foundation for Healthcare Improvement | <https://www.cfhi-fcass.ca/> |
| Institute for Clinical and Evaluative Sciences | <https://www.ices.on.ca/> |

**Appendix-II – Infographic**


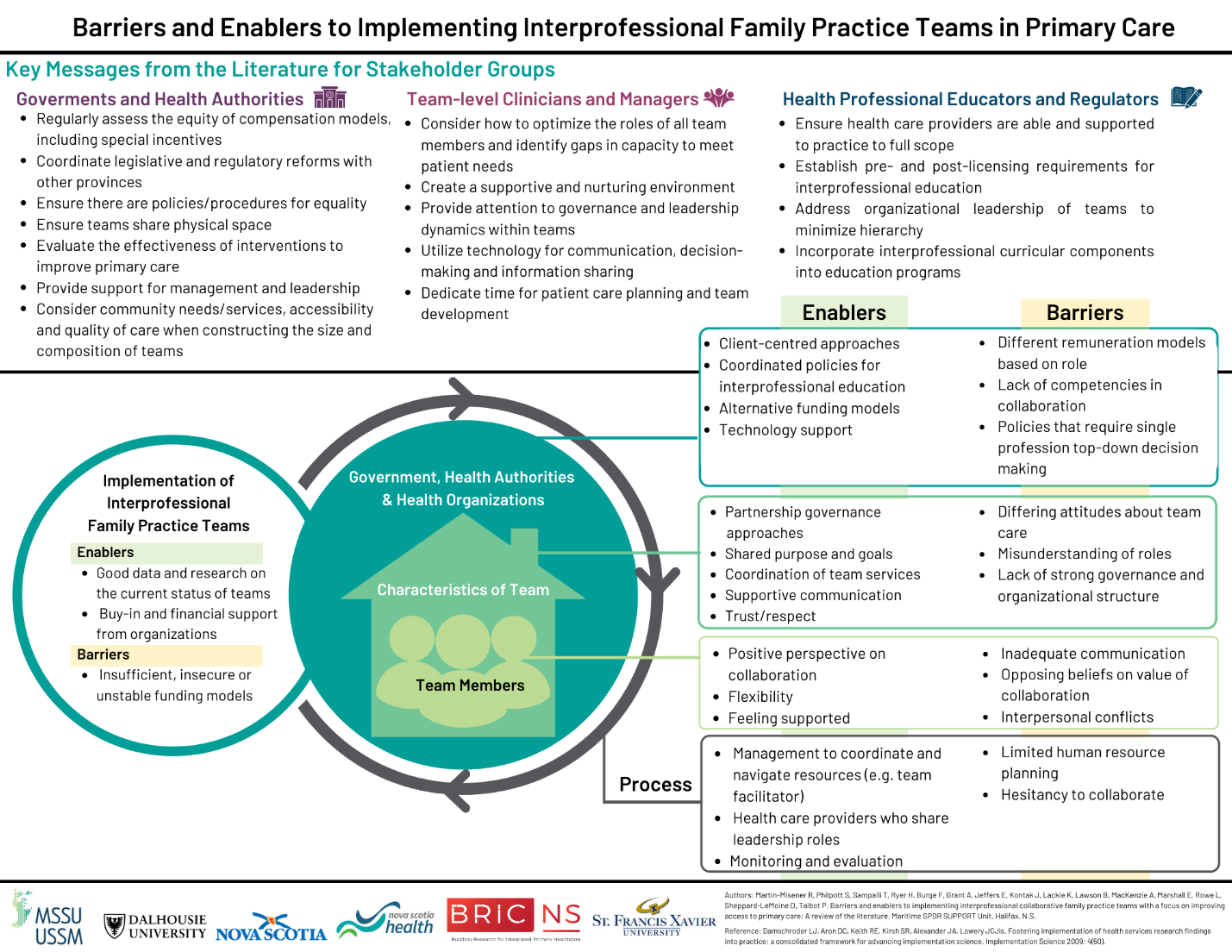

Supplement: Supplementary file 1 — Additional file 1: Appendix-I. Grey literature list of sources. Appendix-II. Infographic [file 12875_2023_2240_MOESM1_ESM.docx]
